# RELATIONSHIP BETWEEN PHYSICAL AND MENTAL HEALTH WITH SUBJECTIVE COGNITIVE DECLINE AMONG OLDER ADULTS IN PUERTO RICO

**DOI:** 10.1093/geroni/igac059.2475

**Published:** 2022-12-20

**Authors:** Thomas Buckley

**Affiliations:** University of Pittsburgh, Pittsburgh, Pennsylvania, United States

## Abstract

Worse physical and mental health are risk factors for cognitive decline in older adults. In Puerto Rico, existing healthcare services are lacking, further exacerbating this risk. This study examines how mental and physical health factors affect subjective cognitive decline for older adults in Puerto Rico. Data comes from the 2020 Behavioral Risk Factor Surveillance System, restricted to adults age 60+ residing in Puerto Rico (n = 1603). Subjective cognitive decline was measured with two dichotomous variables (no/yes): increases in confusion or memory loss and difficulty making decisions in the past year. Multivariate logistic regression models were run for each outcome variables. Predictor variables were number of days in past month with poor mental health, diagnosis of depression or mood disorder, self-rated health, and access to healthcare services, along with covariates. Higher number of days with poor mental health (B = 2.44, p < .001), diagnosis of depression or mood disorder (B = 3.76, p < .001), and cost barriers to accessing healthcare (B = 2.90, p = .002) were associated with increased odds of increased confusion or memory loss. Significant predictors of increased odds of decision-making difficulty included higher number of days of poor mental health (B = 1.88, p < .001), diagnosis of depression or mood disorder (B = 3.80, p < .001), and worse self-rated health (B = 2.13, p < .001). To promote better cognitive health, intervention efforts should focus on those with poor mental and physical health, including identify strategies to improve access to healthcare services.

